# Specific regulation of PRMT1 expression by PIAS1 and RKIP in BEAS-2B epithelia cells and HFL-1 fibroblasts in lung inflammation

**DOI:** 10.1038/srep21810

**Published:** 2016-02-25

**Authors:** Li Liu, Qingzhu Sun, Rujuan Bao, Michael Roth, Bo Zhong, Xi Lan, Jia Tian, Qirui He, Dongmin Li, Jian Sun, Xudong Yang, Shemin Lu

**Affiliations:** 1 Department of Biochemistry and Molecular Biology, School of Basic Medical Sciences, Xi’an Jiaotong University Health Science Center, Xi’an, Shaanxi 710061, P.R. China; 2 Key Laboratory of Environment and Genes Related to Diseases (Xi’an Jiaotong University), Ministry of Education, P.R. China; 3 Pneumology, Department of Biomedicine & University Hospital Basel, University of Basel, Hebelstrasse 20, 4031-Basel, Switzerland

## Abstract

Protein arginine methyltransferase 1 (PRMT1) catalyzes methylation of histones and other cellular proteins, and thus regulates gene transcription and protein activity. In antigen-induced pulmonary inflammation (AIPI) PRMT1 was up-regulated in the epithelium, while in chronic AIPI, increased PRMT1 shifted to fibroblasts. In this study we investigated the cell type specific regulatory mechanism of PRMT1. Epithelial cells and fibroblasts were stimulated with IL-4 or IL-1β. Gene and protein expression were determined by RT-qPCR, immunohistochemistry staining and Western blotting. Signaling pathway inhibitors, siRNAs and shRNA were used to determine the regulatory mechanism of PRMT1. The results showed that IL-4 up-regulated PRMT1 through STAT6 signaling in epithelial cells, while IL-1β regulated PRMT1 through NF-κB in fibroblasts. The NF-kB inhibitor protein RKIP was highly expressed in epithelial cells and blocked IL-1β induced PRMT1 up-regulation; while the STAT6 inhibitor protein PIAS1 was expressed in fibroblasts and suppressed IL-4 induced PRMT1 expression. Furthermore, IL-4 stimulated epithelial cells to release IL-1β which up-regulated PRMT1 expression in fibroblasts. In conclusion, the inhibitor proteins RKIP and PIAS1 regulated the cell type and signaling specific expression of PRMT1. Thus PRMT1 expression in structural lung cells in asthma can be considered as potential target for new therapeutic intervention.

Protein arginine methylation is catalyzed by a family of intracellular enzymes termed protein arginine methyltransferases (PRMT) and is a novel posttranslational protein modification that plays a pivotal role in intracellular signaling, DNA repair, RNA processing, protein-protein interaction and regulation of gene expression. Thereby PRMT1 controls cell differentiation, proliferation, migration and apoptosis and it is implicated that PRMT1 contributes to cardiovascular and pulmonary diseases^1^.

PRMTs are classified as either type I or type II enzymes. Type I PRMTs catalyze the formation of asymmetric dimethylarginine, while types II PRMTs generate symmetric dimethylarginine residues^2^. PRMT1 was the first enzyme of the type I PRMT family which was linked to signal transduction^3^. In our previous study, we have elucidated that IL-4 up-regulated PRMT1 expression in the rat airway epithelium where it increased eotaxin-1 expression and this mechanism was confirmed in a human epithelial cell line. Importantly, pulmonary inflammation waned after inhibiting PRMT activity by AMI-1, which is a pan-PRMT inhibitor^4^. In addition, we showed that PRMT1 expression shifted from the airway epithelium to sub-epithelial fibroblasts when the disease progressed from the acute to the chronic phase. This observation implied that PRMT1 has distinct functions at different disease stages in antigen-induced pulmonary inflammation (AIPI)^5^, thus it may present a novel therapeutic target for asthma.

In the healthy lung the airway epithelium functions a barrier separating the inhaled air from the lung tissue, and there is evidence that the epithelium directly responds to inhaled environmental pro-inflammatory or allergic factors acting as an immune regulator through the secretion of cytokines, chemokines, growth factors, anti-microbial peptides, and recruitment of leukocytes^6^. When the epithelium repair is in-completed, chronic wound repair may take place, and a range of additional growth factors and cytokines are produced which activate the sub-epithelial fibroblasts leading to augmented airway remodeling^7^.

Our previous data clearly demonstrated that PRMT1 participates in both the inflammation and remodeling process in asthma^4^,^5^. In early inflammation, IL-4 increased PRMT1 expression mainly in epithelial cells attracting eosinophil infiltration and exacerbated inflammation in acute AIPI. However, in chronic AIPI, PRMT1 expression was observed mainly in sub-epithelial fibroblasts. Interestingly, PRMT1 expression did not show any significant increase after IL-4 stimulation in fibroblasts. However, few studies investigated the detailed molecular regulatory mechanism of PRMT1 and the involvement of signal pathways and transcription factors controlling PRMT1 expression.

In allergic asthma, Th2 cells drive pulmonary inflammation by activating sub-epithelial fibroblasts which are the major source of extracellular matrix in the interstitial connective tissue of the airways, and thereby contribute to fibrotic changes in the airway wall^8^. Th2 cells release cytokines, prominently IL-4 and IL-13, which activate the signal transducer and activator of transcription-6 (STAT6) proteins^9^, and several counteracting mechanisms have been described in tumorigenesis and immune response^10^,^11^. The transcriptional activity of STAT proteins was down-regulated by the protein inhibitor of activated STAT (PIAS) with a specific interaction of PIAS1, PIAS3 and PIASx with STAT1, STAT3 and STAT4, respectively^12^,^13^,^14^. In addition, PIASy also interacted with STAT1^15^. However, up to date none of the PIAS has been shown to affect STAT6.

Moreover, there are numerous transcription factors, including p53, YY1, and NF-κB, that contribute to the recruitment of PRMTs to various gene promoters^16^. NF-κB is a dimeric transcription factor which is composed of two members of the Rel family of DNA-binding proteins^17^,^18^ and it is activated by various stimuli, such as TNF-α, IL-1β, and PDGF-BB, as well as by bacterial lipopolysaccharide (LPS), viruses (HIV-1, HTLV-1), and exogenous stress factors such as UV, γ-irradiation, and hypoxia^19^. NF-κB activity can be reduced by Raf kinase inhibitor protein (RKIP) which is a scaffold protein facilitating the assembly of a multi-protein complex^20^.

There is no information about the role of STAT6 and NF-κB regarding the cell type specific expression of PRMT1 from lung epithelia to fibroblasts in different stages of AIPI. In this study we found that PRMT1 is regulated by the IL-4/STAT6 signal pathway in epithelial cells, while in fibroblasts PRMT1 is activated by IL-1β through NF-κB. Importantly, we discovered that two signaling inhibitor proteins lead to this cell type specific expression of PRMT1 during different pulmonary inflammation stages.

## Results

### PRMT1 expression is up-regulated by IL-4 in epithelial cells and by IL-1β in fibroblasts

**Figure 1:**
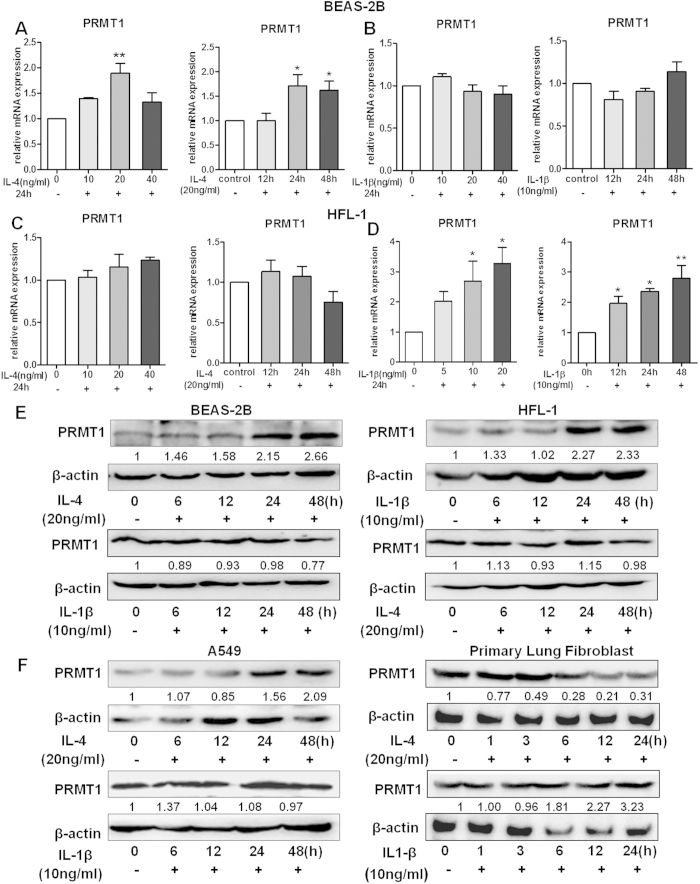
Expression of PRMT1 in BEAS-2B and HFL-1 with IL-4 and IL-1β stimulation. BEAS-2B and HFL-1 cells were stimulated with IL-4 and IL-1β at different doses and time points, and PRMT1 mRNA expression was detected after the stimulation (**A–D**). With 20 ng/ml IL-4 stimulation and 10 ng/ml IL-1β stimulation in BEAS-2B and HFL-1 cells, the protein expression of PRMT1 was measured at different time points after the stimulation (**E**). The relative expression of PRMT1 after stimulation with IL-4 and IL-1β in A549 cells and human primary fibroblasts at different time points was determined by Western blotting (**F**). All the mRNA expression was determined by RT-qPCR analysis, and GAPDH expression was used to normalize the expression level. Western blotting was shown as representative image and density under the band was measured (ImageJ software) and normalized to β-actin. The results were expressed as mean ± S.E.M of triplicates from three independent experiments and analyzed by One-way ANOVA test. * and ** represent *P* < 0.05 and *P* < 0.01 between indicated groups and control group.

To elucidate the different regulatory mechanism in epithelial cells and fibroblasts, IL-4 and IL-1β were used to simulate the BEAS-2B epithelial cell line and the HFL-1 fibroblast cell line. As shown in Fig. 1A, IL-4 increased PRMT1 mRNA expression in BEAS-2B cells after stimulation. IL-1β, however, did not have a stimulating effect in BEAS-2B cells (Fig. 1B). On the contrary, PRMT1 mRNA did not significantly increase after IL-4 stimulation, neither with a series of concentrations nor at different time points in HFL-1 cells (Fig. 1C). However, IL-1β induced PRMT1 mRNA expression in HFL-1 fibroblasts in a dose and time dependent pattern (Fig. 1D). These cell type and stimulus specific effects of PRMT1 regulation were confirmed by protein expression (Fig. 1E). Also, IL-4 and IL-1β were used to simulate the A549 epithelial cell line and human primary lung fibroblasts and Western blotting showed similar results as BEAS-2B cells and HFL-1 cells (Fig. 1F). As we reported earlier^4^,^5^, eotaxin1 and CCR3 were the downstream target genes of IL-4 up-regulated PRMT1 in epithelial cells, while COX2 and VEGF were regulated by PRMT1 in fibroblasts. As shown in Supplementary Fig. 1, the expression of eotaxin1 and CCR3 were up-regulated after IL-4 stimulation in BEAS-2B cells, while the expression of COX2 and VEGF did not change (Supplementary Fig. 1A). Additionally, in fibroblasts, the expression of COX2 and VEGF increased after IL-1β stimulation, while eotaxin1 expression did not increase, and CCR3 was not expressed in fibroblasts (Supplementary Fig. 1B).

### Stimulus and cell type specific response of PRMT1 gene promoter activity differs in epithelial cells and fibroblasts

**Figure 2:**
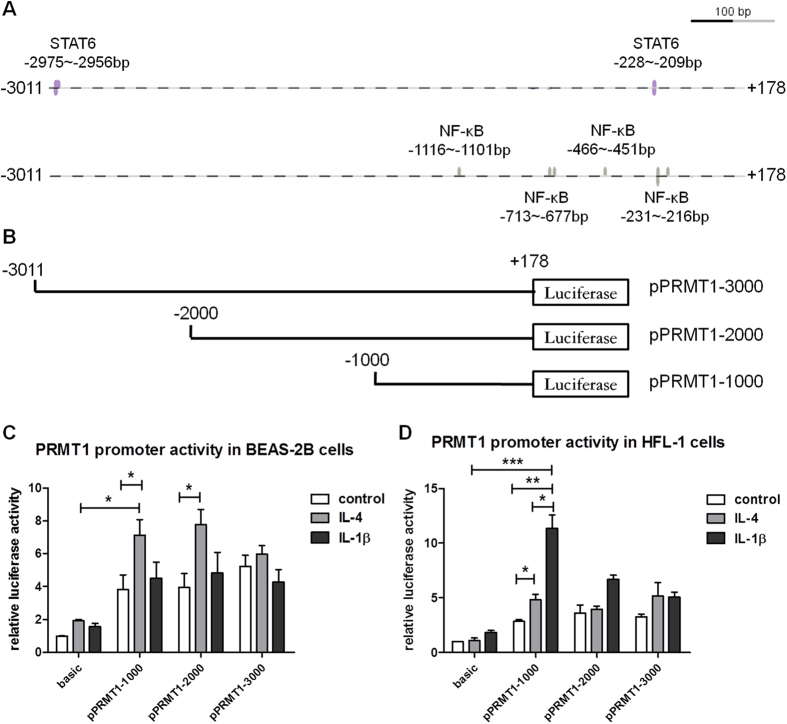
Activation of *PRMT1* gene promoter in BEAS-2B cells and HFL-1 cells stimulated with IL-4 or IL-1β. The interaction sites of STAT6 and NF-κB on PRMT1 gene promoter were predicted (**A**). The schematic diagram of serial 5′-flanking region of *PRMT1* gene promoter (**B**). The relative luciferase activity of three regions of *PRMT1* gene promoter in BEAS-2B or HFL-1 cells were detected by the dual-luciferase reporter assay after transfection pPRMT1 for 48h with or without IL-4 or IL-1β incubated for 24 h (**C,D**). The experiment was performed in quintuplicate and values represent the average of three different experiments after normalization against Renilla activity. The data were expressed as mean ± SEM and analyzed by One-way ANOVA test. *, ** and *** represent *P* < 0.05, *P* < 0.01 and *P* < 0.001 between indicated groups and control group.

Genomatix software analysis predicted that the sequence of the *PRMT1* promoter contains two binding sites for STAT6 and four binding sites for NF-κB. The first binding site of STAT6 of the *PRMT1* promoter was located at bp −227 to −209 and the second one at bp −2974 to −2956. Four binding sites for NF-κB were predicted at bp +200 to −1000 and one at bp −1116 to −1101 (Fig. 2A). To identify the reactive binding sites in the *PRMT1* promoter, we constructed a series of human *PRMT1* promoter clones from −3000 to +200bp, named pPRMT-3000, pPRMT1-2000, and pPRMT1-1000 (Fig. 2B). In BEAS-2B cells the *PRMT1* promoter constructs pPRMT1-1000 and pPRMT1-2000 were significantly activated by IL-4 but not by IL-1β; while, pPRMT1-1000 was activated by IL-4 and IL-1β in HFL-1 cells (Fig. 2C,D).

### The expression of PRMT1 in HFL-1 cells increases after exposure to IL-4 stimulated epithelial cell medium

**Figure 3:**
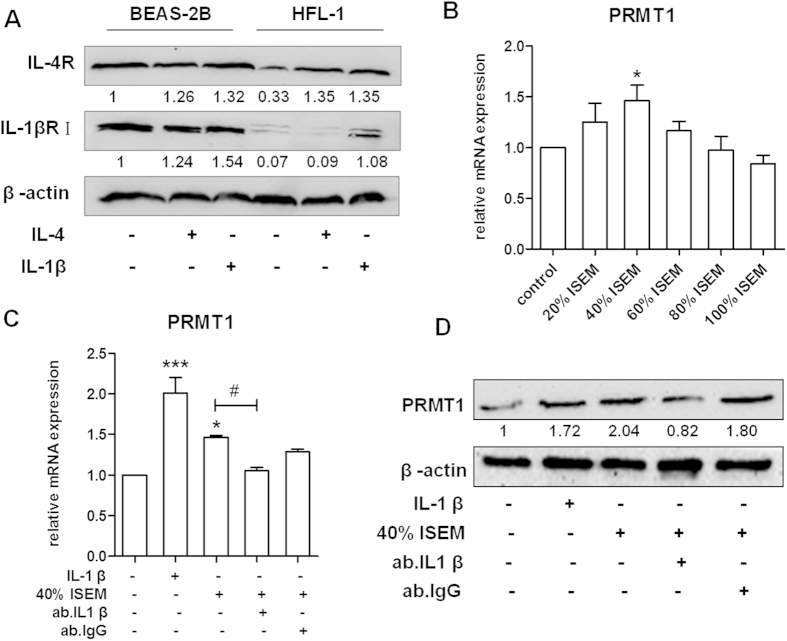
Expression of IL-4 receptor and IL-1β receptor I in BEAS-2B and HFL-1 cells and expression of PRMT1 after stimulation with ISEM in HFL-1 cells. The expression of IL-4 receptor and IL-1β receptor I were detected by Western blotting with or without IL-4 and IL-1β stimulation in BEAS-2B and HFL-1cells (**A**). The supernatant from IL-4-stimulated BEAS-2B cells (ISEM) was accumulated after 48 hours stimulation and 5% FBS was added to the culture supernatant to replenish the consumption by epithelial cells. Then ISEM was used to stimulate HFL-1 cells. The expression of PRMT1 in HFL-1 cells was determined by RT-qPCR after 24h stimulation by different concentrations of ISEM (20%, 40%, 60%, 80%, 100%) (**B**). IL-1β, 40% ISEM, and IL-1β antibody were used to stimulate HFL-1 cells and the expression of PRMT1 was detected by RT-qPCR and Western blotting (**C,D**). Western blotting was shown as representative image and density under the band was measured (ImageJ software) and normalized to β-actin. The results were expressed as mean ± S.E.M of triplicates from three independent experiments and analyzed by One-way ANOVA test. *,^#^ and ** represent *P* < 0.05 and *P* < 0.01 between indicated groups and control group.

To explore how PRMT1 is regulated by different cytokines and signal pathways in epithelial cells and fibroblasts, we determined the expression of IL-4 and IL-1β receptors in BEAS-2B and HFL-1 cells. Western blotting showed that the IL-4 receptor (IL-4Rα) was expressed in both BEAS-2B and HFL-1 cells and was up-regulated after either IL-4 or IL-1β stimulation (Fig. 3A). Furthermore, the IL-1β receptor (IL-1βRI) was expressed on both BEAS-2B and HFL-1 cells. It is noteworthy that the expression of IL-1βRI was lower in fibroblasts compared to epithelial cells and increased significantly after IL-1β stimulation (Fig. 3A).

To further explore the possible link between the two cell types on the regulation of PRMT1 expression, increasing concentrations of IL-4 stimulated epithelial cell medium (ISEM) were added to HFL-1 cells and the expression of PRMT1 was increased significantly by 40% ISEM at 24h (Fig. 3B). To verify whether IL-1β is the component in ISEM which up-regulates PRMT1 expression in HFL-1 cells, a neutralizing antibody against IL-1β was added, and an antibody to IgG was used as an isotype control. The data showed that IL-1β depletion attenuated the expression of PRMT1 on both mRNA and protein level (Fig. 3C,D).

### The IL-4/STAT6 pathway is activated in BEAS-2B cells but not in HFL-1 cells

**Figure 4:**
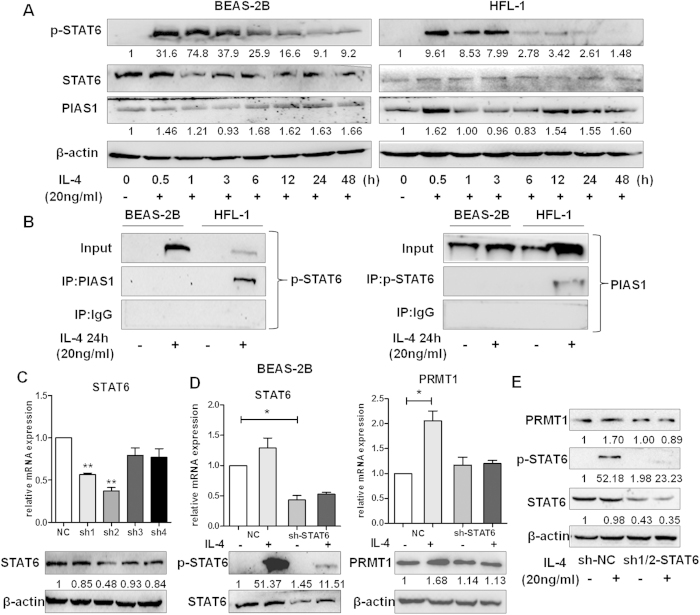
Activation of IL-4/STAT6 pathway is in BEAS-2B cells rather than in HFL-1 cells. The BEAS-2B and HFL-1 cells were incubated with IL-4 (20 ng/mL) for the indicated time, and the expression and phosphorylation of STAT6 and the expression of PIAS1 were detected by Western blotting. The interaction of p-STAT6 and PIAS1 was detected by Co-IP with anti-p-STAT6, anti-PIAS1 antibody or anti-IgG antibody (negative control group) in BEAS-2B and HFL-1 cells with or without IL-4(20 ng/mL) incubated for 24 h (**B**). The relative mRNA and protein expressions of STAT6 were detected by RT-qPCR and Western blotting after transient transfection with shRNA-STAT6 vector or shRNA-NC vector as control in BEAS-2B cells for 24h (**C**).The mRNA and protein expression of STAT6 and PRMT1 and phosphorylation of STAT6 in BEAS-2B cells with stable expression of sh2-STAT6 vector and sh-NC vector was detected with or without IL-4 stimulation (**D**). sh1-STAT6 and sh2-STAT6 vectors were mixed at the ratio of 1:1 to knockdown the STAT6 expression and protein expression of STAT6 and PRMT1 and phosphorylation of STAT6 in BEAS-2B cells was detected with or without IL-4 stimulation (**E**). Western blotting was shown as representative image and density under the band was measured (ImageJ software) and normalized to β-actin. The results were expressed as mean ± S.E.M of triplicates from three independent experiments and analyzed by One-way ANOVA test. * and ** represent *P* < 0.05 and *P* < 0.01 between indicated groups and control group.

With IL-4 stimulation, phosphorylation STAT6 reached a maximum between 30 min and 1 hour both in BEAS-2B and HFL-1 cells (Fig. 4A). Since IL-4 did not show any stimulatory effect on PRMT1 expression in fibroblasts, we further investigated the underlying signal transduction. The results showed that IL-4 stimulation increased PIAS1 protein levels in BEAS-2B and HFL-1 cells (Fig. 4A). To further determine the action of PIAS1, co-IP assay with p-STAT6 was performed and the result revealed that PIAS1 and p-STAT6 formed a complex in HFL-1 cells, but not in BEAS-2B cells (Fig. 4B).

We further aimed to verify the function of STAT6 on PRMT1 expression in IL-4 simulated epithelial cells. Thus, BEAS-2B cells were transfected with either shRNA-STAT6 vector or shRNA-NC as control. Both RT-qPCR and Western blotting results showed that the sh2-STAT6 vector knocked down STAT6 expression effectively (Fig. 4C). Next, we established stable sh-STAT6 knockdown cells, and observed that IL-4 stimulation for 24h neither induced the phosphorylation of STAT6 nor PRMT1 significantly compared to shRNA-NC transfection (Fig. 4D). In order to avoid off-target effects of shRNA and confirm the effect of STAT6 knockdown, additional experiments were performed by using a mix of sh1-STAT6 and sh2-STAT6 vectors at the ratio of 1:1 to knockdown the STAT6 expression and Western blotting showed similar results as stable sh-STAT6 knockdown cells (Fig. 4E).

### The IL-1β/NF-κB pathway is activated in HFL-1 cells but not in BEAS-2B cells

**Figure 5:**
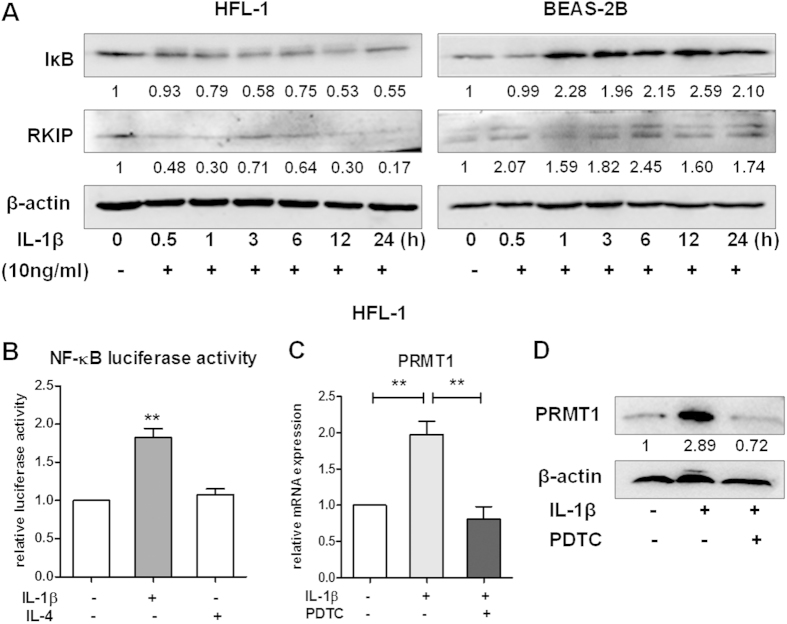
Activation of IL-1β/NF-κB pathway is in HFL-1 cells rather than in BEAS-2B cells. The BEAS-2B and HFL-1 cells were incubated with IL-1β (10 ng/mL) for the indicated time, and total protein samples were analyzed by Western blotting (**A**). The relative luciferase activity of NF-κB in HFL-1 cell was detected by dual-luciferase reporter assay after transfection of pNF-κB for 48h with IL-1β or IL-4 stimulation in quintuplicate (**B**). The relative mRNA and protein expressions of PRMT1 were detected by RT-qPCR and Western blotting with or without IL-1β (10 ng/mL) and PDTC (10 nM) incubated for 24 h (**C,D**). Western blotting was shown as representative image and density under the band was measured (ImageJ software) and normalized to β-actin. The results were expressed as mean ± S.E.M of triplicates from three independent experiments and analyzed by One-way ANOVA test. The results were expressed as mean ± S.E.M and analyzed by One-way ANOVA test. * and ** represent *P* < 0.05 and *P* < 0.01 between indicated groups and control group.

In order to explore the IL-1β stimulated signaling pathway regulating PRMT1 expression in epithelial cells and fibroblasts, BEAS-2B and HFL-1 cells were stimulated for 30min, 1h, 3h, 6h, 12h or 24h. The expression of IκB and RKIP were detected by Western blotting. In fibroblasts, IL-1β degraded IκB; however, in BEAS-2B cells IL-1β increased the expression of IκB (Fig. 5A). Interestingly, IL-1β increased RKIP, a NF-κB pathway inhibitor, in BEAS-2B cells and decreased it in HFL-1 cells (Fig. 5A). Further analysis using NF-κB luciferase activity assay showed that IL-1β significantly increased NF-κB activity in HFL-1 cells compared to unstimulated cells and IL-4 treated fibroblasts (Fig. 5B). To confirm the regulatory role of the NF-κB signal pathway in IL-1β regulation of PRMT1, fibroblasts were incubated with PDTC, a NF-κB inhibitor, which decreased the IL-1β stimulated PRMT1 both on the mRNA and protein level (Fig. 5C,D). Furthermore, VEGF and COX2 were also decreased by PTDC in HFL-1 cells (Supplementary Fig. 2).

### Cell type specific expression of RKIP and PIAS1 in epithelial cells and fibroblasts accounts for the cell type specific expression of PRMT1

**Figure 6:**
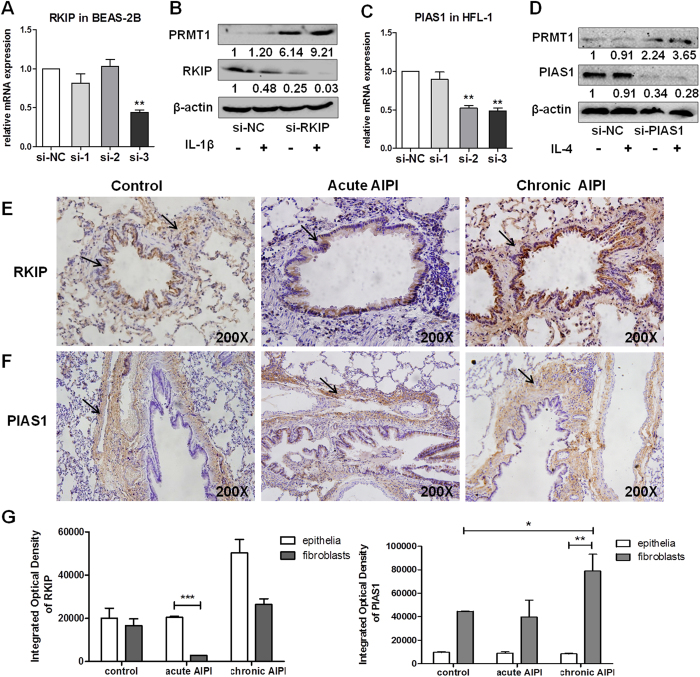
Effects of knockdown RKIP or PIAS1 in BEAS-2B and HFL-1 cells and expression and location of RKIP and PIAS1 in AIPI lungs. BEAS-2B cells were transfected with 40nM si-RKIP or si-NC, while HFL-1 cells were transfected with 40nM si-PIAS1 or si-NC for 24h. The mRNA expression of RKIP and PIAS1 were detected by RT-qPCR (**A,C**). The si-RKIP-3 was used to knockdown RKIP expression and IL-1β was added to stimulate the BEAS-2B cells. The mix of si-PIAS1-2 and si-PIAS1-3 was used to knockdown PIAS1 expression and IL-4 was added in HFL-1 cells. The protein expressions of RKIP, PIAS1 and PRMT1 were detected after transfection and stimulation (**B,D**). RKIP and PIAS1 were detected by immunohistochemical staining. Representative images of RKIP (**E**) and PIAS1 (**F**) protein expression in control rat lungs (left panel), acute (middle panel) and chronic AIPI rat lungs (right panel) were stained with anti-RKIP (1:1000) and anti-PIAS1 antibody (1:100). IOD (integrated optical density) of RKIP and PIAS1 was determined by Image-Pro Plus 6.0 software to estimate the expression of protein in epithelium and sub-epithelium (n = 6 for each group) (**G**). Western blotting was shown as representative image and density under the band was measured (ImageJ software) and normalized to β-actin. The results were expressed as mean ± S.E.M of triplicates from three independent experiments and analyzed by One-way ANOVA test. * and ** represent *P* < 0.05 and *P* < 0.01 between indicated groups and control group.

To explore the cell type specific role of RKIP and PIAS1, both inhibitor proteins were silenced by corresponding siRNAs in BEAS-2B or in HFL-1 cells, respectively. In BEAS-2B cells, si-RKIP-3 was the most efficient siRNA and was used for all further experiments (Fig. 6A). Western blotting showed that PRMT1 protein expression was rescued after knockdown of RKIP in IL-1β stimulated epithelial cells (Fig. 6B). Additionally, Western blotting of the phosphorylation of STAT6 in si-RKIP treated cells showed that IL-1β is not a strong inducer of STAT6 phosphorylation (Supplementary Fig. 3).

In HFL-1 cells, si-PIAS1-2 and si-PIAS1-3 showed an equal knock-down effect thus equal amounts of si-PIAS1-2 and si-PIAS1-3 were mixed for all following experiments (Fig. 6C). The expression of PRMT1 was increased in HFL-1 cells treated with si-PIAS1 as compared to si-NC treated cells (negative control). In HFL-1 cells treated with si-PIAS1 and stimulated with IL-4 the expression of PRMT1 was up-regulated compared to the control-group (Fig. 6D).

### Cell type specific expression pattern of RKIP and PIAS1 is observed in E3 rat lungs with AIPI

We next determined the expression of RKIP and PIAS1 *in vivo*, by immunohistochemical staining in rat AIPI lungs. The results showed that RKIP was mainly expressed in airway epithelium and to a lesser extend in fibroblasts in control and animals with chronic AIPI (Fig. 6E). In lungs with acute AIPI, RKIP was mainly expressed in the airway epithelium (Fig. 6E). In contrast, PIAS1 was equally expressed in the sub-epithelial fibroblasts in control and acute AIPI lungs, while it was increased in fibroblasts of rats with chronic AIPI (Fig. 6F,G).

## Discussion

In this study, we observed that IL-4 stimulated PRMT1 by STAT6 pathway in epithelial cells, while IL-1β stimulated PRMT1 via NF-κB in fibroblasts. In addition, our *in vitro* data suggested that IL-4 stimulated epithelial cells released IL-1β which in turn elevated PRMT1 production by fibroblasts. Furthermore, an increased expression of the STAT inhibitor protein PIAS1 was observed, which formed a complex with p-STAT6, thereby inhibiting STAT6 signaling in fibroblasts, but not in epithelial cells. In epithelial cells the high expression of IκB and RKIP prevented IL-1β stimulation of PRMT1 expression. These findings were confirmed *in vivo* by immunohistochemistry showing PIAS expression in sub-epithelial fibroblasts and RKIP expression in the airway epithelium.

PRMTs have essential regulatory functions as transcription factor co-regulators. *In vitro* studies revealed that angiotensin-II induced PRMT1 expression and endothelial cell activation, thereby generating reactive oxygen species (ROS)^21^. Extracts of nerve growth factor treated PC12 cells contained elevated PRMT1 activity, compared to untreated cells^22^. PRMT1 also played an important role in gene activation and metamorphosis induced by ligand activated thyroid hormone receptor^23^,^24^. In our previous study, we elucidated that PRMT1 expression was up-regulated in acute AIPI rat lungs and was stimulated by IL-4 in airway epithelial cells^4^. Furthermore, our data showed that PRMT1 expression shifted from the epithelium to sub-epithelial fibroblasts where it was significantly up-regulated in rat lungs with chronic AIPI^5^. However, the differences of the regulatory mechanism of PRMT1 in different cell types needed to be unveiled.

There is strong evidence that epithelium dysfunction is a cause of inflammatory lung disorders, and sub-epithelial fibroblasts are the major source of extracellular matrix in airway wall interstitial connective tissue in asthma^25^. IL-4 is one of the most important pro-inflammatory cytokines driving airway inflammation and IL-1β is a potent pro-inflammatory mediator that exacerbates parenchymal-cell injury^8^. Our data clearly showed that IL-4 up-regulated the expression of PRMT1 in epithelial cells and IL-1β increased its expression in fibroblasts. In addition, four down-stream genes of PRMT1, eotaxin-1 and CCR3 in epithelial cells, and COX2 and VEGF in fibroblasts, were increased by IL-4 or IL-1β in the same cell type specific pattern as PRMT1. These data indicated that PRMT1 was regulated by different signal pathways in different airway cell types.

Regarding PRMT1 promoter function studies using the *Xenopus* PRMT1 promoter indicated that thyroid hormone (T3) up-regulated PRMT1 through c-Myc, a transcription factor associated with stem cells and cell proliferation^26^. However, to our knowledge, there is no report on transcription factors regulating the human PRMT1 promoter in the lung. Therefore, we have cloned the human promoter (−3011/+178) upstream the PRMT1 transcription start site (TSS) and sequence analysis by Genomatix software of the −3011/+178bp region revealed two putative bindings sites for STAT6 and four binding sites for NF-κB. By using 5′-serial deletion reporter gene constructs, we showed that the region from bp −1000 of the human PRMT1 promoter was required for the response of BEAS-2B to IL-4 and to IL-1β in HFL-1 cells, which suggested that the PRMT1 promoter −1000/+178 bp was a core promoter region for PRMT1 regulation. In HFL-1 cells the PRMT1 promoter −1000/+178bp was activated by IL-4, but IL-1β was more effective. Additionally, the promoter region −3011/+178bp in BEAS-2B cells, −2000/+178bp and −3011/+178bp in HFL-1 cells, which contain the STAT6 and NF-κB binding sites, seemed unresponsive. From this result, we speculate that a PRMT1 promoter suppressor may exist for these unresponsive regions.

As PRMT1 is induced by different cytokines in lung epithelial cells and fibroblasts, and participates in both inflammation and remodeling in asthma, we investigated the link between these two cell types on PRMT1 expression. The ability of alveolar epithelial cells to release chemotactic factors was recognized in a seminal paper published in 1990, describing the ability of A549 cells to release IL-8 in response to TNF or IL-1β^27^. Andrew *et al.* reported that alveolar type II epithelial cells release IL-1β in response to LPS^28^, and in our previous study we demonstrated that IL-4 stimulated A549 epithelial cells to produce TGF-β, which affected the proliferation of fibroblasts, and elevated the production of PRMT1, COX2 and VEGF^5^. Here we provide evidence that IL-4 stimulated human epithelial cells released IL-1β which then up-regulated PRMT1 expression in fibroblasts. Blocking IL-1β activity in epithelial cell conditioned medium by neutralizing IL-1β antibodies inhibited the increased expression of PRMT1 by fibroblasts.

In various cell types, the transcription factor STAT6 mediates the action of IL-4. Our data showed that IL-4 exposure phosphorylated STAT6 both in BEAS-2B and HFL-1 cells. Moreover, IL-4 phosphorylated STAT6 in HFL-1 cells but did not up-regulate the expression of PRMT1. In this context, PIAS proteins bound specifically to phosphorylated STAT dimers and prevented them from binding to corresponding DNA binding sequences^12^,^29^. Since there is specificity as well as redundancy in PIAS-STAT interactions we aimed to explain why the phosphorylation of STAT6 in HFL-1 cells did not up-regulate the expression of PRMT1. We observed that the STAT inhibitor protein PIAS1 was increased in both BEAS-2B and HFL-1 cells after IL-4 stimulation. Co-IP proved a complex formed by both factors after stimulation in HFL-1 cells but not in BEAS-2B cells. This may explain the different effects of IL-4 on PRMT1 regulation in BEAS-2B and HFL-1 cells. Furthermore, IL-4 did not up-regulate the expression of PRMT1 in shRNA-STAT6 treated cells compared to shRNA-NC treated cells, confirming that PRMT1 is a target of IL-4/STAT6 signaling in epithelial cells.

In addition, we demonstrated that knockdown of PIAS1 rescued the STAT6 activity in HFL-1 cells and increased PRMT1 expression after IL-4 stimulation. Moreover, the expression and location of PIAS1 *in vivo* in AIPI rat lung tissue sections showed that it was highly expressed in sup-epithelial cells, suggesting that IL-4 may have no direct stimulating effect on PRMT1 expression in sub-epithelial fibroblasts. In AIPI lung tissues, PIAS1 was expressed in airway epithelial cells which might represent a negative feedback mechanism of STAT6 signaling; thus our data suggested that the cell type specific IL-4 effects on the expression of PRMT1 required the STAT6 inhibitor protein PIAS1.

Sub-epithelial fibroblasts are important in airway wall remodeling in asthma and their numbers are increased in asthmatic airways^30^. TGF-β, bFGF, GM-CSF, IL-1β, and TNF-α may play pivotal roles in this process, as reviewed by others^31^,^32^. Among those cytokines, IL-1β induced epithelial-mesenchymal transition (EMT) and myofibroblast activation through a TGF-β1 mediated mechanism^33^. In our study, we observed the degradation of the NF-κB inhibitor IκB in HFL-1 cells after IL-1β stimulation, thereby activating the NF-κB signaling. In contrast, there was no degradation of IκB in BEAS-2B cells after IL-1β stimulation, but the expression of RKIP, a NF-κB signaling inhibitor protein, was increased. Interestingly, the expression of RKIP decreased in HFL-1 cells stimulated with IL-1β. These observations may explain the different effects of IL-1β on NF-κB activation and regulation of PRMT1 expression in the two structure forming airway wall cell types. Moreover, in IL-1β stimulated HFL-1 cells the NF-κB inhibitor PDTC reduced the expression of PRMT1 and down-stream genes of PRMT1, VEGF and COX2.

Furthermore, knockdown of RKIP rescued the NF-κB activity in BEAS-2B cells and the PRMT1 expression increased after IL-1β stimulation without phosphorylation of STAT6. Therefore, we considered that PRMT1 up-regulation in si-RKIP treated cells was due to NF-κB activation by IL-1β but not a potentiating effect of IL-1β on top of IL-4. Immunohistochemical staining of RKIP in AIPI rat lungs showed its major expression in bronchial epithelial cells. This observation may explain why IL-1β did not induce PRMT1 expression in BEAS-2B cells.

**Figure 7:**
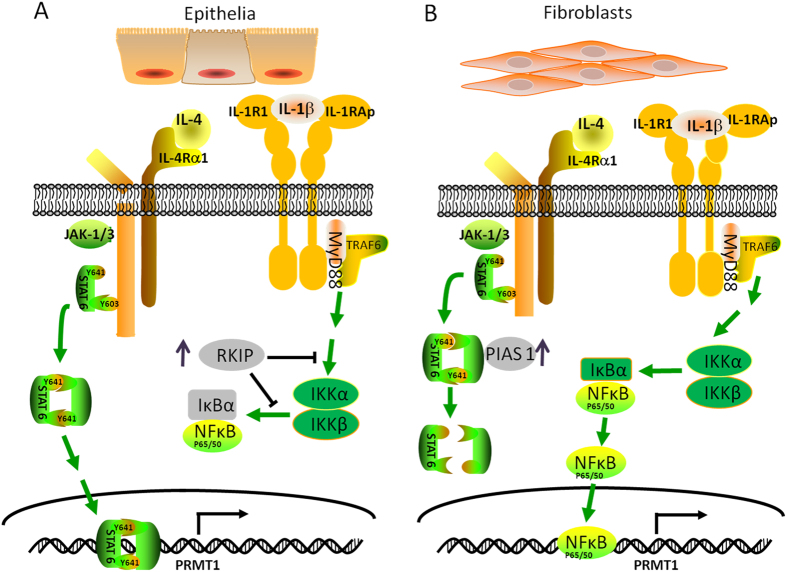
Schematic diagram of cell type specific regulation of PRMT1. In epithelial cells, IL-4 activated STAT6 signaling and then PRMT1 expression was up-regulated, while IL-1β increased RKIP and IκB expression and restrained the NF-κB signaling (**A**). In fibroblasts, up-regulated PIAS1 inhibit STAT6 signal pathway which is integral for IL-4 induced PRMT1 up-regulation, and IL-1β up-regulated PRMT1 expression via NF-κB signaling pathway (**B**). The mechanism explains why in acute asthma, the PRMT1 expression up-regulated in epithelium, while in chronic asthma, PRMT1 is up-regulated in fibroblasts.

In summary, we demonstrated that IL-4 up-regulated PRMT1 expression through STAT6 in epithelial cells, while IL-1β induced PRMT1 in fibroblasts through NF-κB. We also proved that IL-4 stimulated epithelial cells released IL-1β, which in turn up-regulated PRMT1 expression by fibroblasts. The findings revealed two cell type specific regulatory mechanisms on PRMT1 which are mediated by the cell type specific inhibitor proteins PIAS1 and RKIP (Fig. 7). Beside novel information on the cell type specific conditions leading to allergic asthma, our data may provide the basis for three novel therapeutic and diagnostic targets in asthma as PRMT1, PIAS1 and RKIP.

## Materials and Methods

### Cell culture and stimuli

BEAS-2B, a normal human bronchial epithelial cell line, was kindly provided by Professor Wei Wang (School of Public Health, Zhenzhou University, China) and the cells were grown in RPMI-1640 (Invitrogen, Grand Island, NY) supplemented with 12% NCS (newborn calf serum) (Beyotime, Beijing, China).

HFL-1, a normal human fibroblast line, was bought from the Type Culture Collection of the Chinese Academy of Sciences, Shanghai, China. The cells were grown in F12K (Invitrogen, Grand Island, NY) supplemented with 10% FBS (fetal bovine serum) (Hyclone, Logan, UT).

Human A549 alveolar epithelial-like cells (A549) were cultured in RPMI 1640 (Invitrogen, Grand Island, NY) supplemented with 10% FCS (HyClone, Logan, UT).

Lung tissue specimens were obtained from the clinic of pneumology (University Hospital Basel, Switzerland), with the approval of the local Ethical Committee (EK:05/06) and written consent of each tissue donor (n = 3). Primary human lung fibroblasts were isolated from tissues of lung biopsies as previously described^34^ and cells were propagated under standard cell culture conditions (37 °C, 100% humidity, 5% CO2, 95% air).

Human recombinant IL-4 and IL-1β (PeproTech, New Jersey, USA) were added into the wells (12-well plates and 6-well plates) at increasing concentrations (0, 10, 20, 40ng/ml). PDTC, a NF-κB inhibitor (Beyotime, Beijing, China), was used at 10μM in HFL-1 cells which were pre-incubated for 24h.

IL-4 stimulated epithelial cell medium (ISEM) was obtained from IL-4 stimulated BEAS-2B cells (48hours) collected and centrifuged (10 min) to remove cells and cell debris, and 5% FBS was added to the culture supernatant to replenish the consumption by epithelial cells and then stimulate HFL-1 cells at a series of concentration with F12K basic medium (20%, 40%, 60%, 80%, 100%). 10ng/ml IL-1β cytokine (PeproTech, New Jersey, USA) or 1μg/ml anti-IL-1β antibody or anti-IgG (Abcam, Cambridge, UK) was mixed with ISEM to investigate IL-1β effects on the regulation of PRMT1.

### Stably expressed clone screening and RNA interference

For transfection, BEAS-2B cells were seeded in 12-well plates and grown to 80% confluence before the pGPU6/GFP/Neo shRNA-STAT6 vector or shRNA-NC vector (GenePharma, Shanghai, China) was transfected using LipofectamineTM 2000 (Invitrogen, Carlsbad, USA) according to the manufacturer’s instructions. The cells were incubated with the recombinant complexes for 5h, and then replaced with the complete medium. RPMI 1640 medium with 400ng/ml G418 was added into the cells 24h after transfection and the medium was replaced every 2 days over a period of 2 weeks, until the stable expression clones were screened by limiting dilution analysis. The most efficient shRNA-STAT6 vector (sh2-STAT6) had the following sequence: 5′-CACCGGCTGATCATTGGCTTCATCATTCAAGAGATGATGAAGCCAATGATCAGCCTTTTTTGA-3′.

The small interfering RNAs (siRNA) were designed and synthesized by Genechem (Shanghai, China). BEAS-2B cells and HFL-1 cells were transfected with siRNA according to manufacturer’s protocols. The sequence of the most effective small interference RNA for RKIP (si-RKIP-3) was: sense, GCUAUGUCUGGCUGGUUUATT; antisense, UAAACCAGCCAGACAUAGCTT. The sequence for the si-PIAS1 was: sense, CCAGCAACUUUGUCUCCAUTT, antisense, AUGGAGACAAAGUUGCUGGTT; and for si-PIAS1–3: sense, GCAGCCUGGUUUCUUCCAATT, antisense, UUGGAAGAAACCAGGCUGCTT. The siRNA sequences were mixed with Lipofectamine 2000 (Invitrogen, Carlsbad, CA) and used at a final concentration of 40 nM for transfecting the cells, with for 24 h. The gene expression was determined by RT-qPCR.

### Human *PRMT1* promoter cloning and reporter gene assay

The human *PRMT1* promoter fragments were generated by PCR with the primer sequences provided in Table 1. The PCR products were cloned into the pGL3-basic vector with double restriction enzyme sites (sense *Kpn* I, antisense *Bgl* II) which were then confirmed by PCR, double restriction enzyme reaction, and DNA sequencing.

Cells were seeded in 48-well plates and transfected with 270ng pGL3-pPRMT1 plasmid and 30ng *Renilla* luciferase reporter plasmid for 24h, and then stimulated with IL-4 or IL1-β or sham for 24h. The pNF-κB luciferase reporter plasmid (Beyotime, Beijing, China) at 270ng and 30ng *Renilla* luciferase reporter plasmid were transfected into HFL-1 for 24h and then stimulated with IL-4 or IL1-β or sham for 24h. Luciferase activity in cell lysates was measured by dual-luciferase reporter assay kit (Promega, Madison, USA) 48h after transfection by Thermo Scientific Luminoskan Ascent Microplate Luminometer (Thermo, USA).

### RNA quantitations

The mRNAs expression was determined by real-time quantitative PCR (RT-qPCR), performed with the iQ5 real-time PCR detection system (Bio-Rad, CA, USA) using a SYBR Premix Ex Taq TM II (Roche, Basel, Switzerland) and the relative gene expression was normalized to GAPDH. The information of primers is shown in Table 2.

### Western blotting

BEAS-2B cells, A549 cells, HFL-1 cells or primary lung fibroblasts were lysed with RIPA cell lysis buffer (Beyotime, Beijing, China). The lysates were centrifuged at 12000 rpm (15 min) and the supernatant was collected for Western blotting. The protein concentration was quantified by BCA (Themo, Beijing, China). Equal amounts (20μg) of denatured protein were separated by SDS–PAGE, and then electro-transferred onto PVDF membranes. The following primary antibodies were used to incubate overnight in 4 °C: anti-pSTAT6 antibody (1:500 dilution, Abcam, Cambridge, UK), anti-STAT6 antibody (1:500 dilution, Abcam, Cambridge, UK), anti-PRMT1 antibody(1:500 dilution, Abcam, Cambridge, UK), anti-IκB antibody (1:1000 dilution, CST, Boston, USA), anti-COX2 antibody (1:300 dilution, Abcam, Cambridge, UK), anti-PIAS1 antibody (1:100 dilution, CST, Boston, USA), anti-RKIP antibody (1:250 dilution, CST, Boston, USA) and anti-β-actin antibody (1:2000 dilution, CST, Boston, USA).

### Co-Immunoprecipitation assay

For co-immunoprecipitation (Co-IP) BEAS-2B cells with or without IL-4 stimulation and HFL-1 cells with or without IL1-β stimulation grown in 100mm plates were lysed in cell lysis buffer. Each lysate was divided into three parts, and 1μg of anti-p-STAT6 antibody or anti-PIAS1 antibody was incubated overnight at 4 C with one of the lysates, anti-IgG antibody served as negative control. Immune complexes were precipitated by Protein-A/G Agarose (Beyotime, Shanghai, China) at 4 C for 4h. After five washes with cold 1×PBS, an equal volume of 2× loading buffer was added to the complexes. Next, denatured protein samples were separated by SDS-PAGE for Western blotting with respective antibodies to p-STAT6, PIAS1 or IgG as described above.

### Lung histology and immunohistochemistry staining

Lung tissues sections from AIPI rats were obtained from previous experiments^3^. For immunohistochemical staining, tissue sections were incubated with anti-PIAS1 antibody (1:100, Abcam, Cambridge, UK) or anti-RKIP antibody (CST, Boston, USA) in PBS at 4 °C overnight, followed by 2-step plus Poly-HRP anti-goat IgG detection kit (ZSGB-BIO, Beijing, China) as described previously^3^ (n = 6 for each group). The experimental procedures were approved by the Institutional Animal Ethics Committee of Xi’an Jiaotong University; the methods were carried out in “accordance”with the approved guidelines.

### Statistical analysis

All data are presented as mean ± SEM from three independent experiments performed in triplicate or quadruplicate. All Western blotting data are representative of three independent experiments, and the protein band density was measured by ImageJ software and normalized to β-actin. The mean value of the protein bands is shown underneath the corresponding protein bands. Statistical comparison was performed by One-way ANOVA test with Tukey post hoc testing between multiple treatment groups. *P*-value < 0.05 was considered as statistically significant.

## Additional Information

**How to cite this article**: Liu, L. *et al.* Specific regulation of PRMT1 expression by PIAS1 and RKIP in BEAS-2B epithelia cells and HFL-1 fibroblasts in lung inflammation. *Sci. Rep.* **6**, 21810; doi: 10.1038/srep21810 (2016).

## Supplementary Material

Supplementary Information

## Figures and Tables

**Table 1:** Primer information for construction of PRMT1 promoter vectors.

Gene	Species	Sequence(5′-3′)	Size (bp)	annealing temperature (°C)
pPRMT1-3000	*Homo sapiens*	Forward CGGGGTACCCAAGAGCCTGTCTCCTGGA Reverse GGAAGATCTTCCAAGCGCTCACCTCCA	3101	56
pPRMT1-2000	*Homo sapiens*	Forward CGGGGTACCCAGCTGGGCTCCTCTTGC Reverse GGAAGATCTTCCAAGCGCTCACCTCCA	1988	56
pPRMT1-1000	*Homo sapiens*	Forward CGGGGTACCCGCCCGAACCCTATTGTA Reverse GGAAGATCTTCCAAGCGCTCACCTCCA	1011	54

**Table 2:** Primer information for RT-qPCR.

Gene	Species	Sequence(5′-3′)	Size (bp)	annealing temperature (°C)
PRMT1	*Homo sapiens*	Forward TTGACTCCTATGCCCACT Reverse CCACATCCAGCACCACC	126	62
eotaxin1	*Homo sapiens*	Forward TTCTGTGGCTGCTGCTCATCG Reverse GGCAACTAGTCGCTGAAGGG	125	63
CCR3	*Homo sapiens*	Forward GGCAATGTGGTGGTGGTGATG Reverse GGAAGGGTGACGAGGAAGAGC	113	62
COX2	*Homo sapiens*	Forward GTGCAACACTTGAGTGGCTAT Reverse GCAATTTGCCTGGTGAATGAT	249	60
VEGF	*Homo sapiens*	Forward CGGCGAAGAGAAGAGACACATTG Reverse CGGGAAGGGAAGGGAAGGAC	224	60
STAT6	*Homo sapiens*	Forward GAAGTGCCCGCTGAGAAAGG Reverse ACCAGACCCCACAGAGACAT	106	60
GAPDH	*Homo sapiens*	Forward CGGCAAGTTCAACGGCACAG Reverse GAAGACGCCAGTAGACTCCACGAC	148	60
PIAS1	*Homo sapiens*	Forward ACAGTGCGGAACTAAAGCAAA Reverse AACCGCCGCCTATAGAGTTC	187	60
RKIP	*Homo sapiens*	Forward ACGCCCACCCAGGTTAAGA Reverse TGCCCTTCATGTTGACCACC	160	60
