# Supplementary material for: Specific regulation of PRMT1 expression by PIAS1 and RKIP in BEAS-2B epithelia cells and HFL-1 fibroblasts in lung inflammation
Source: Sci Rep. 2016 Feb 25;6:21810. doi: 10.1038/srep21810 (PMC4766407; doi:10.1038/srep21810)
Supplement: Supplementary Information [file srep21810-s1.doc]

**Specific regulation of PRMT1 expression by PIAS1 and RKIP in BEAS-2B epithelia cells and HFL-1 fibroblasts in lung inflammation**

Li Liu^1, 2#^, Qingzhu Sun^1, 2, 3#^, Rujuan Bao^1, 2^, Michael Roth^3^, Bo Zhong^1, 2^, Xi Lan^1, 2^, Jia Tian^1, 2^, Qirui He^1, 2^ , Dongmin Li^1, 2^, Jian Sun^1, 2^, Xudong Yang^1, 2*^, Shemin Lu^1, 2*^

**One Sentence Summary**: Cell type specific mechanism of PRMT1 in epithelium and fibroblast

1 Department of Biochemistry and Molecular Biology, School of Basic Medical Sciences, Xi’an Jiaotong University Health Science Center, Xi’an, Shaanxi 710061, P. R. China;

2 Key Laboratory of Environment and Genes Related to Diseases (Xi’an Jiaotong University), Ministry of Education, P. R. China;

3 Pneumology, Department of Biomedicine & University Hospital Basel, University of Basel, Hebelstrasse 20, 4031-Basel, Switzerland

^#^ These authors contributed equally to this work.

* Address correspondence to: Prof. Shemin Lu, or Dr. Xudong Yang, Department of Biochemistry and Molecular Biology, School of Basic Medical Sciences, Xi’an Jiaotong University Health Science Center, Xi’an, Shaanxi 710061, P. R. China, Tel: +86-29-82657764, Fax: +86-29-82657764

E-mail address: lushemin@mail.xjtu.edu.cn or yangxd@mail.xjtu.edu.cn

**Supplementary Figure 1**

**
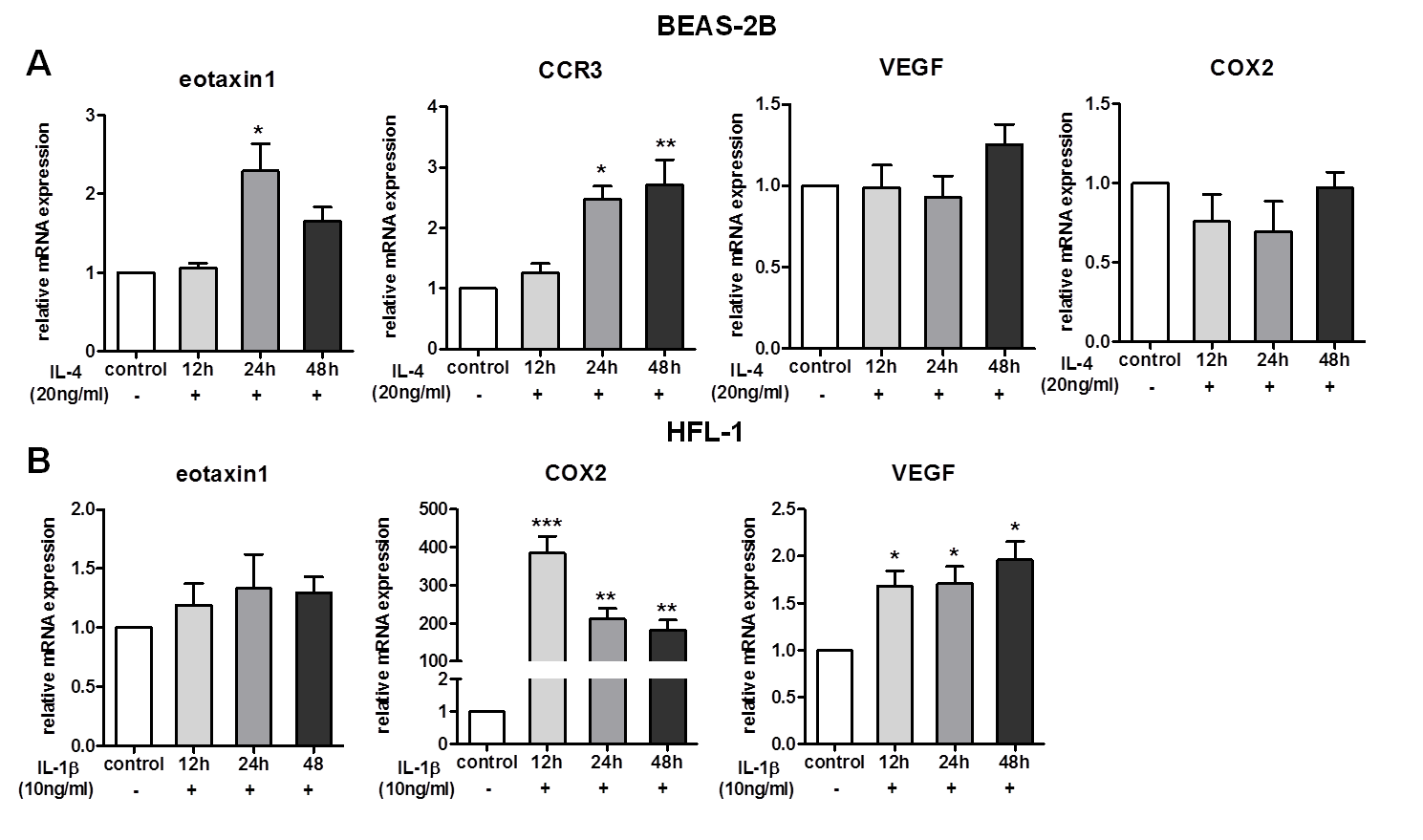
**

**Supplementary Figure 1 The relative expression of eotaxin1, CCR3, COX2 and VEGF in BEAS-2B and HFL-1 cells.** After the stimulation of IL-4 in BEAS-2B or IL-1β in HFL-1 cells, mRNA expression was measured by RT-qPCR and GAPDH expression was used to normalize the expression level (A, B). The results were expressed as mean±S.E.M of triplicates from three independent experiments and analyzed by One-way ANOVA test. * and ** represent *P*＜0.05 and *P*＜0.01 between indicated groups and control group.

**Supplementary Figure 2**

**
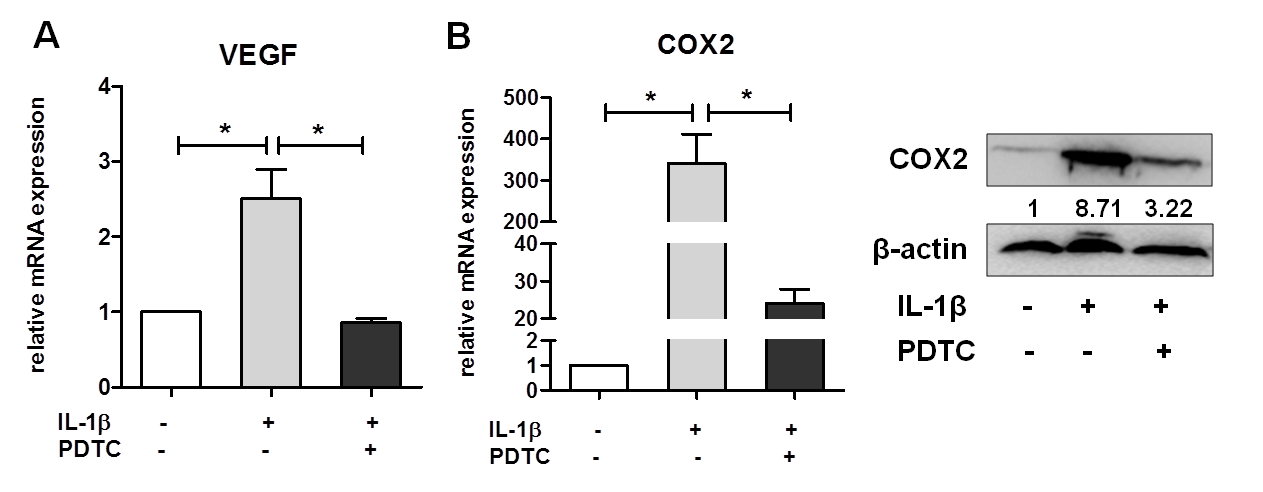
**

**Supplemetary Figure 2 The relative mRNA and protein expression of VEGF and COX2.** RT-qPCR and Western blotting were performed in HFl-1 cells with or without IL-1β (10ng/mL) and PDTC (10nM) incubated for 24h(C, D). Western blotting were shown as representative image and density under the band was measured (ImageJ software) and normalized to β-actin. The results were expressed as mean±S.E.M of triplicates from three independent experiments and analyzed by One-way ANOVA test. * and ** represent *P*＜0.05 and *P*＜0.01 between indicated groups and control group.

**Supplementary Figure 3**

**
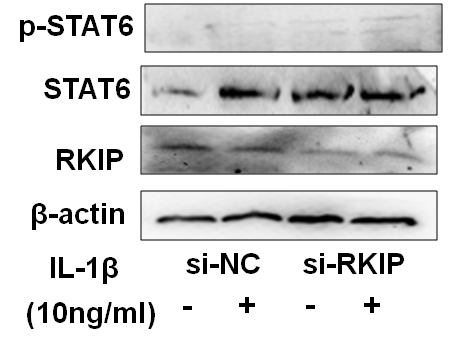
**

**Supplementary Figure 3 Representative Western blotting of RKIP knockdown effects in BEAS-2B cells.** BEAS-2B cells were transfected with 40nM si-RKIP or si-NC. The si-RKIP-3 was used to knockdown RKIP expression and IL-1β was added to stimulate the BEAS-2B cells for 6h after transfection. The protein expression of RKIP, p-STAT6 andSTAT6 was detected 6 hours after transfection and stimulation.
